# Serum hyaluronic acid concentration predicts the progression of joint space narrowing in normal knees and established knee osteoarthritis – a five-year prospective cohort study

**DOI:** 10.1186/s13075-015-0793-0

**Published:** 2015-10-10

**Authors:** Eiji Sasaki, Eiichi Tsuda, Yuji Yamamoto, Shugo Maeda, Ryo Inoue, Daisuke Chiba, Hiroshi Fujita, Ippei Takahashi, Takashi Umeda, Shigeyuki Nakaji, Yasuyuki Ishibashi

**Affiliations:** Department of Orthopaedic Surgery, Hirosaki University Graduate School of Medicine, 5 Zaifu-cho, Hirosaki, Aomori 036-8562 Japan; Department of Social Medicine, Hirosaki University Graduate School of Medicine, Hirosaki, Japan; Glycoconjugate Research Center, Kurihama Plant, Seikagaku Corporation, Yokosuka, Japan

## Abstract

**Introduction:**

Serum hyaluronic acid (sHA) is a serum biomarker for knee osteoarthritis (OA). Although sHA concentration is elevated in patients with knee OA, the relationship between serum concentration and disease progression remains unclear. We examined the relationship between sHA concentration and radiographic progression of knee OA in a cohort of individuals followed for 5 years.

**Methods:**

We prospectively enrolled 444 individuals and measured their sHA concentrations at baseline. Anterior-posterior weight bearing knee radiographs were obtained at baseline and the 5-year endpoint. Osteoarthritic knee changes were classified according to Kellgren–Lawrence (KL) grade, and joint space narrowing (JSN) was measured using a Knee Osteoarthritis Computer-Aided Diagnosis (KOACAD) system. Correlations between sHA concentration, progression in KL grade, and JSN were assessed using regression models, taking into account potentially confounding factors.

**Results:**

OA progressed from KL grades 0 or 1 in 129 of the 323 knees, and from KL grades 2 or 3 in 61 of the 119 knees. Higher sHA concentrations were correlated with KL grade progression (p = 0.004). The mean JSN, as assessed by KOACAD over 5 years, was 0.23 ± 0.55 mm, and sHA concentration was positively correlated with progression of JSN in KL grades 0 or 1 (p = 0.021) and KL grades 2 or 3 (p = 0.008) knees.

**Conclusion:**

Serum HA concentration was positively correlated with progression of KL grade. sHA was also positively correlated with progression of JSN in knees with and without OA, suggesting that sHA concentration may be a useful predictor of knee OA progression.

## Introduction

Osteoarthritis (OA) is a common cause of pain and disability in elderly people [1]. The prevalence of radiographic knee OA among adults over 40 years old in Japan was recently reported as up to 42.0 % in men and 62.4 % in women [2]. Knee OA imposes a heavy burden of pain and disability, including high costs of treatments, decreased productivity, and absence from work. Consequently, although OA should be diagnosed as soon as possible to begin treatment, the current diagnostic tools are inadequate. The gold standard method of diagnosis has long been plain radiography, but the sensitivity and specificity of that technique have been questioned [3]. Magnetic resonance imaging (MRI) may allow for earlier OA diagnosis because it is capable of detecting cartilage damage, small osteophytes, subchondral bone changes, and synovitis in the presence or absence of symptoms [4–6]. Nevertheless, MRI is expensive, time consuming, and contraindicated in some patients.

Serum biomarkers are a potentially useful alternative tool besides conventional diagnostic imaging examination. Biomarkers allow disease activity to be objectively evaluated, are easily measured in office-based practices, and, in rheumatoid arthritis and osteoporosis, can help patients understand their condition. Several biomarkers are known to be correlated with the extent of OA on radiography of the knee and have thus been proposed as diagnostic tools [7–10]. Among such biomarkers, serum hyaluronic acid (sHA) is particularly promising. Several previous cross-sectional studies have reported that measuring sHA concentration may be useful for not only diagnosing knee OA, but also identifying disease duration, severity, and the extent of OA-related knee pain [11–15]. Nonetheless, the currently used biomarkers remain insufficiently discriminating for predicting the prognosis of OA [7]. The concentration of sHA is thought to reflect the extent of synovitis, which is present at the onset of OA and accelerates disease progression by producing proteases and cytokines [16–18]. Therefore, sHA may have potential as a prognostic indicator of progressive knee OA, but the relationship between sHA and knee OA has only been examined in a few longitudinal studies [19, 20].

In the present study, we determined the relationship between sHA concentration and radiographic changes in the knee in a 5-year prospective patient cohort. To objectively assess knee radiography, the knee osteoarthritis computer-aided diagnosis (KOACAD) system was used to measure joint space narrowing (JSN) and osteophyte formation, and the knees were grouped according to the Kellgren–Lawrence (KL) grade [21, 22]. We hypothesized that sHA concentration is correlated with the severity of knee OA and can predict the development and progression of OA as seen on radiography of the knee.

## Methods

### Participants

The participants had volunteered for the Iwaki Health Promotion Project, which is a community-based preventative medicine program that aims to improve average life expectancy by performing general health checkups and prophylactic interventions, as previously described [12, 13]. All participants gave their written informed consent, and the study was conducted with the approval of the ethics committee of the Hirosaki University School of Medicine.

**Fig. 1:**
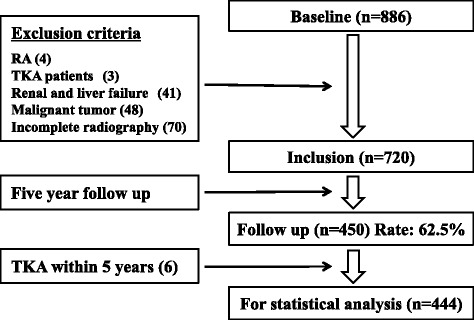
Number of participants in the cohort enrolled, excluded, and followed up at 5 years. The values in parentheses indicate the number excluded. *RA* rheumatoid arthritis, *TKA* total knee arthroplasty

A total of 866 volunteers (325 men and 561 women) from approximately 12,000 eligible residents enrolled in the project in 2008 (Fig. 1). The participants were recruited using mass media advertisements and public health nurses. Of these, 70 declined radiographic examination, and 96 were excluded because of a history of total knee arthroplasty, rheumatoid arthritis, renal failure, liver failure, malignant tumors, or having returned incomplete questionnaires. After 5 years, 450 of the participants underwent the endpoint checkup (follow-up rate 62.5 %): of these, 6 (1.3 %) had undergone total knee arthroplasty. In total, 444 participants (158 men and 286 women) were included in the analysis. The 270 participants who dropped out over the 5 years led to a low follow-up rate. However, because participation in this observational cohort was part of a general health checkup and entirely voluntary, we could not force them to participate in the endpoint checkup. However, no significant differences were found in the baseline data between the followed participants and those who dropped out (Table 1). Additional questionnaires on lifestyle habits were administered, including questions about alcohol consumption, cigarette smoking, and fitness habits. The presence of these habits was defined as drinking, smoking, or taking exercise one or more days per week. These habits were included in the statistical analysis as a categorical variable (yes/no). Height and weight were measured and recorded by project team staff, and body mass index (BMI) was calculated.

**Table 1:** Baseline demographic data and Kellgren − Lawrence (KL) grades of the participants who were followed up and those who dropped out

	Dropped out (n = 270)	Followed up (n = 444)
Age, years	56.9 ± 14.4	55.4 ± 12.0*
Female, %	61.4	64.4
Body mass index, kg/m^2^	22.9 ± 3.2	23.0 ± 3.1
Serum HA, ng/ml	67.2 ± 48.2	64.7 ± 46.2
Smoking habit, %	18.3	17.5
Drinking habit, %	41.1	42.6
Fitness habits, %	22.3	31.6
KL grade 0/1, n	177	323
KL grade 2, n	67	91
KL grade 3, n	22	28
KL grade 4, n	4	2

The values are baseline demographic data for the participants who dropped out and those who were followed up. Patients who underwent total knee arthroplasty during the 5 years are not included in this table. *P value <0.05 (considered statistically significant) for comparison of the two groups *HA* hyaluronic acid

Data were mean ± SD

### Serum HA assay

Fasting venous blood specimens were taken from all participants at baseline in the early morning for biochemical examination. Serum was obtained by centrifugation, separated into 0.7 ml aliquots, and stored at −80 °C for later analysis. The sHA concentration was determined using the Hyaluronan Assay Kit (Seikagaku Corporation, Tokyo, Japan). The intra-assay and inter-assay coefficients of variation were 3.2 % and 5.0 %, respectively, when a pooled sample of normal human sera was tested.

### Radiographic examination of the knee

Experienced radiologic technicians took the weight-bearing and anterior-posterior radiographs of both knees with foot map positioning, at baseline and at the endpoint. The beam was positioned parallel to the floor with no angle and aimed at the joint space, following Oka’s technique for using the KOACAD system (INOTECH corporation, Hiroshima, Japan) [21]. The images were converted into Joint Photographic Experts Group (JPEG) format files. The severity of OA in each knee was classified by two trained orthopedic surgeons (YI and RI) as KL grade 0 to 4 using the KL radiographic atlas [22]. The interclass correlation coefficient between the two surgeons was 0.815. The surgeons were blinded to the sequence in which the radiographs were acquired and the clinical status of the participants. A diagnosis of knee OA was made in participants with KL grades of 2, 3 or 4 in the most affected knee, and the same knee was examined at the endpoint. Development of knee OA was defined as knees with KL grades 0 or 1 at baseline changing to KL grades 2, 3, or 4 over the 5 years. Progression of knee OA was defined as knees with KL grade 2 changing to KL grade 3 or 4, and as knees with KL grade 3 changing to KL grade 4, over 5 years. The joint space width (JSW) at the narrowest point in the medial or lateral compartment of the femoro-tibial joints and the total osteophyte areas on the proximal tibia and distal femur were calculated from calibrated radiographs using the KOACAD system. Joint space narrowing (JSN) was defined as a reduction in the JSW over 5 years. The rate of JSN and changes in osteophyte formation from baseline to the 5-year endpoint were calculated. The KOACAD system is a fully automated system for quantifying the major features of knee OA on standard radiographs, allowing for an objective, accurate, and straightforward assessment of the structural severity of knee OA in general clinical practice. It has proven to be highly reliable and reproducible, with an intraclass correlation coefficient of 1.00 based on the validation study that included 1,979 knee radiographs [21]. After defining a region of interest (ROI) in the KOACAD system that included the tibiofemoral joint space, a vertical neighborhood difference filter was applied to identify locations with high absolute values of difference of scales. Within the ROI, the outline of the femoral condyle was defined as the upper rim of the joint space. The outlines of the anterior and posterior margins of the tibial plateau were similarly drawn, and the middle line between the two outlines was defined as the lower rim of the joint space. The minimum JSW was further determined as the minimum vertical distance in the joint space area. To measure osteophyte area, the medial outlines of the tibia and femur from the inflection point were extended upward to the joint level and the area that became medially prominent over the smoothly extended outline was designated as the osteophyte area [21].

### Statistical analysis

The Mann–Whitney *U* test and Chi square test were used to compare the demographic data between the participants who were followed and those who dropped out. The Kolmogorov–Smirnov test was used to verify that the sHA concentration and rate of JSN data were normally distributed, which they were (*p* = 0.200). Spearman’s correlation coefficient was calculated to examine the association between sHA concentration, rate of JSN, and risk factors for OA based on age, sex, and BMI. The parameters determined from the knee radiographs (JSW, JSN, rate of JSN, osteophyte area, and osteophyte formation for each KL grade) were compared using analysis of variance (ANOVA). The relationship between sHA and radiographic change in the knee was investigated using cross-sectional analysis at baseline and longitudinal analysis over 5 years. For the cross-sectional analysis, the mean sHA concentrations were compared using ANOVA and Tukey’s post hoc test. Logistic regression analysis was performed using progression from KL grades 0 or 1 and progression from KL grades 2 or 3 as dependent variables and age (per year of age), sex (female versus male), BMI (per 1 kg/m^2^), and sHA concentration (per 1 ng/ml) as independent variables, which were adjusted for cigarette smoking, alcohol consumption, and fitness habits. Similarly, linear regression analysis was performed using rate of JSN or osteophyte formation as the dependent variable and age, sex, and BMI as the independent variables, which were adjusted for cigarette smoking, alcohol consumption, and fitness habits. Finally, to estimate the predictive cutoff level of sHA concentration for developing knee OA from KL grades 0 or 1 and the progression of knee OA from KL grades 2 or 3, receiver operating characteristic (ROC) analysis was performed. The values of sHA were used as a single variable in this analysis. The false positive fraction was plotted against the 1 − true positive fraction, and the cutoff point was defined as the point of the maximum slope, i.e. the nearest point to true positive. Furthermore, the area under the curve (AUC) was calculated to evaluate the validity of the ROC analysis. Data input and analyses were performed in SPSS version 12.0 J (SPSS Inc., Chicago, IL, USA). *P* values <0.05 were considered statistically significant.

## Results

The mean age of the followed participants at baseline was 55.4 ± 12.0 years (range 25–85 years), which was lower than that of the participants who dropped out (*p* = 0.04). The mean BMI was 23.0 ± 3.1 kg/m^2^ (range 15.6–37.5 kg/m^2^). Women accounted for 64.4 % of the subjects. No significant differences were found for gender (*p* = 0.76), BMI (*p* = 0.97), sHA concentration (*p* = 0.35), KL grade (*p* = 0.13), or lifestyle habits between the followed subjects and those who dropped out (Table 1).

### Serum HA concentration and cross-sectional analysis

**Fig. 2:**
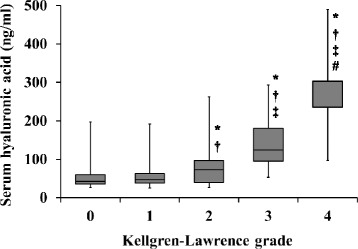
Concentration of serum hyaluronic acid by Kellgren–Lawrence (KL) grade. *P* values <0.05 were found compared with *KL grade 0, ^†^grade 1, ^‡^grade 2, and ^#^grade 3

The mean baseline sHA concentration of the participants who were followed up was 64.7 ± 46.2 ng/ml (range 25.0 − 304.0 ng/ml), which was not significantly different to the concentration in those who dropped out (*p* = 0.353). The serum HA concentration increased with age (correlation coefficient 0.67, *p* <0.001) and BMI (correlation coefficient 0.15, *p* = 0.006), but sHA concentration was not correlated with sex (correlation coefficient 0.03, *p* = 0.595). Cross-sectional analysis showed that the mean sHA concentration increased with the severity of knee OA, and significant differences were found between all of the KL grades, except between KL grades 0 and 1 (Fig. 2).

### Radiographic evaluation of knee OA

There were 323 participants with KL grade 0 or 1, 91 with grade 2, 28 with grade 3, and 2 with grade 4 (Table 2). Radiographic examination at the 5-year endpoint showed that the KL grade in 190 of 443 participants (42.1 %) with KL grades 0, 1, 2, or 3 at baseline had progressed. Osteoarthritic disease was assessed as having developed in 14 knees and progressed in 61 knees.

**Table 2:** Knee osteoarthritis in participants by Kellgren–Lawrence (KL) grade at baseline, and changes over 5 years

Baseline		Endpoint				Development/progression
KL grade	Number	KL grade				
		0/1	2	3	4	
0, 1	323	194	96	33		129
2	91		44	43	4	47
3	28			14	14	14
4	2				2	
Total	444	194	140	90	20	190

The values are the numbers of participants

The KOACAD parameters were determined among all participants and by KL grade. The mean JSW was 3.46 ± 0.82 mm among all participants, and for KL grades 0 through 4 it was 3.9 ± 0.6, 3.5 ± 0.7, 3.2 ± 0.7, 2.5 ± 1.2, and 0.2 ± 0.1 mm, respectively. The mean progression of JSN over 5 years was 0.23 ± 0.55 mm among all participants, and for KL grades 0 through 4 it was 0.2 ± 0.5, 0.1 ± 0.5, 0.3 ± 0.5, 1.0 ± 0.8, and 0 mm, respectively. The mean rate of JSN was 9.31 ± 17.6 % (−18.2 to 100.0 %) among all participants, and for KL grades 0 through 4 it was 3.3, 2.0, 9.4, 50.0, and 0 %, respectively. The mean osteophyte area was 2.05 ± 8.74 mm^2^ among all participants, and for KL grades 0 through 4 it was 1.0 ± 6.7, 1.3 ± 4.7, 1.8 ± 4.5, 12.1 ± 26, and 15.7 ± 22 mm^2^, respectively. The mean osteophyte formation was 1.62 ± 12.89 mm^2^ among all participants, and for KL grades 0 through 4 it was 0.6, 0.5, 3.6, 6.6, and 12.6 mm^2^, respectively.

### Longitudinal analysis

**Fig. 3:**
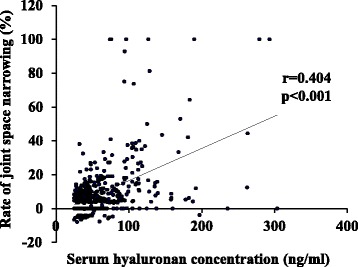
Serum hyaluronic acid concentration versus the rate of joint space narrowing

**Fig. 4:**
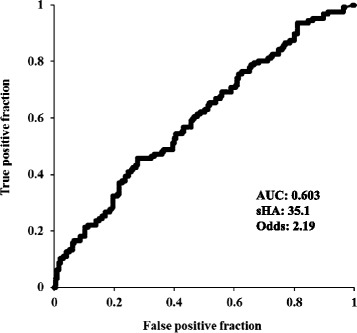
Receiver operating characteristic (ROC) curves for serum hyaluronic acid (*sHA*) and development of knee osteoarthritis from Kellgren−Lawrence (KL) grades 0 or 1. *AUC* area under curve, *Odd*s odds ratio

**Fig. 5:**
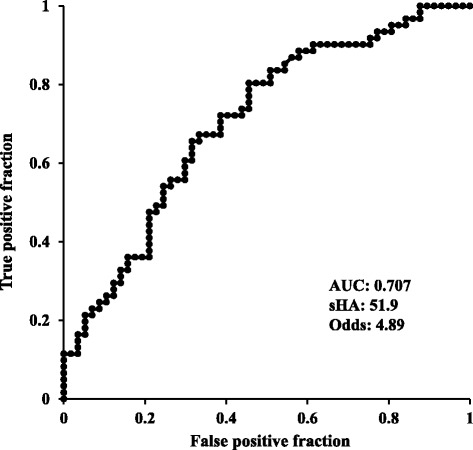
Receiver operating characteristic (ROC) curves for serum hyaluronic acid (*sHA*) and progression of knee osteoarthritis from KL grades 2 or 3. *AUC* area under curve, *Odds* odds ratio

The Spearman’s coefficient for correlation between sHA concentration and the rate of JSN was 0.404 (*p* <0.001) (Fig. 3). Adjusted logistic regression analysis showed that higher sHA concentration was correlated with progression from KL grades 2 or 3 (*p* = 0.004), but not with development from KL grades 0 or 1 (*p* = 0.196; Table 3). A positive correlation was found between being female and the development of OA from KL grades 0 or 1, and aging and higher BMI were correlated with both the development and progression of OA. However, significant correlation was found between higher sHA concentration and the progression of JSN in knees with KL grades 0 or 1 at baseline (*p* = 0.021) and with KL grades 2 or 3 at baseline (*p* = 0.008; Table 4). ROC analysis showed that the sHA cutoff value for predicting the development from KL grades 0 or 1 was 35.1 ng/ml (AUC 0.603), and the odds ratio at that point was 2.19 (Fig. 4). The sHA cutoff value for predicting the progression from KL grades 2 or 3 was 51.9 ng/ml (AUC 0.707), and the odds ratio at that point was 4.89 (Fig. 5).

**Table 3:** Relationship between serum hyaluronic acid concentration and changes in Kellgren–Lawrence (KL) grade

	Development from KL grade 0/1				Progression from KL grade 2/3			
	*β*	*P* value	Odds	95 % CI	*β*	*P* value	Odds	95 % CI
Females	0.87	0.005	2.39	1.27−4.24	0.54	0.472	1.71	0.40–7.42
Age	0.04	0.014	1.04	1.01–1.07	0.07	0.016	1.08	1.01–1.14
BMI	0.16	0.001	1.18	1.07–1.30	0.24	<0.001	1.28	1.11–1.46
sHA	0.01	0.196	1.01	0.99–1.02	0.02	0.004	1.02	1.01–1.03

Logistic regression analysis of KL grades 0 or 1 and KL grades 2 or 3 at baseline. Dependent variables: development from KL grades 0 or 1 and progression from KL grades 2 or 3. Independent variables: age, sex, body mass index (*BMI*), and serum hyaluronic acid (*sHA*) concentration, which was adjusted for lifestyle parameters. *KL grade β* standardized coefficient

**Table 4:** Correlations between baseline serum hyaluronic acid concentrations and the KOACAD parameters from knee radiographs

	KL grade 0/1		KL grade 2/3	
	*β*	*P* value	*β*	*P* value
Rate of joint space narrowing	0.15	0.021	0.24	0.008
Osteophyte formation	−0.05	0.422	0.25	0.017

Linear regression analysis was performed on knees with radiographic Kellgren–Lawrence (*KL*) grades 0 or 1 and KL grades 2 or 3 at baseline. Dependent variables: rate of joint space narrowing and osteophyte formation in KL grades 0 or 1 and KL grades 2 or 3. Independent variables: age, sex, body mass index, and serum hyaluronic acid (sHA) concentration, which was adjusted for lifestyle parameters. The values indicate the correlation with sHA concentration. *KOACAD* knee osteoarthritis computer-aided diagnosis, *β* standardized coefficient

## Discussion

The results of the present longitudinal study show that sHA concentration at baseline was positively correlated with the development or progression of KL grade. Furthermore, sHA concentration at baseline was correlated with JSN in both normal and severely osteoarthritic knees, as assessed by radiograph. These findings are clinically relevant and suggest that sHA concentration reflects not only the severity, but also the likelihood of progression of knee OA. Thus, sHA concentration may be useful as a prognostic marker for predicting JSN, though not for predicting osteophyte formation. Because OA is a long-term, chronic disease, it is difficult to fully understand the relationship between sHA and the incidence of OA based on only a 5-year observation. However, the present study is the first report showing that an sHA cutoff value of 51.9 ng/ml was predictive of OA progression based on ROC analysis. Concentrations of sHA >51.9 ng/ml indicate a high risk of progression of knee OA. We hope that these results are applied to the screening during conventional knee examinations or the clinical risk evaluation for progressive OA after knee injury or surgery. Further clinical studies are needed to determine whether sHA can predict the incidence of OA, which was not determined in the present study.

The long-term cohort with a large sample size used in the present study made it possible to estimate the sHA cutoff value as 51.9 ng/ml, which was indicative of a five-fold increase in the risk of JSN over 5 years. Several studies have reported sHA concentration cutoff values as biomarkers for knee OA. For example, Kaneko et al. suggested cutoff values of sHA to define the presence of knee OA using confidence intervals matched with the KL grades in a clinical cross-sectional study [23]. However, the present study is the first to estimate the cutoff value predictive of the progression of knee OA based on a longitudinal cohort. Unfortunately, the reliability of the cutoff value for the development of knee OA was low because the AUC was 0.603. On the other hand, the cutoff value of the progression of OA in those with moderate knee OA was valid and reliable based on its higher AUC. Thus, we believe that this cutoff value should be useful during screening for abnormal knee conditions or as an additional evaluation for the risk of OA progression when used in combination with conventional imaging tools.

The mechanisms underpinning the phenomenon of elevated sHA concentration in progressive knee OA are thought to be mechanical stress and synovitis with cartilage degeneration [24]. OA is characterized by focal damage to the articular cartilage centered on the load-bearing areas, osteophyte formation at the joint margins, subchondral bone changes, and synovitis [17]. Synovitis is present at the onset of OA [18] and results in the production of hyaluronic acid (HA) and pro-inflammatory cytokines [25]. In addition, synovitis activates fibroblasts, which promote the production of other pro-inflammatory cytokines such as tumor necrosis factor-α and interleukin-1β [17]. These cytokines promote matrix metalloproteinase production by fibroblasts, which degrade the articular cartilage matrix [16]. Thus, the presence of synovitis is thought to contribute to the progression of knee OA. Ayral et al. suggested that synovitis was also predictive of subsequent chondropathy [26]. Severe OA is a risk factor for acceleration of the progression of knee OA [26], which broadly reflects our findings that sHA concentration was correlated with disease progression in the longitudinal and cross-sectional analyses.

Although previous studies have described the relationship between sHA and progression of knee OA, the data were limited. Pavelka et al. reported that the non-adjusted correlation coefficient between sHA and JSN over 2 years was 0.56 based on 89 patients with knee OA [19]. Similarly, Sharif et al. reported that the sHA concentration measured in 12 patients with 2 mm JSN over 5 years was higher than that in patients without progressive JSN [20]. However, these studies had relatively small sample sizes, selection biases derived from focusing only on patients with knee OA, and used only JSN as the sole outcome measure; therefore the relationship between sHA and the pathogenesis of OA was not fully examined. In contrast, we elucidated these relationships in a general population of individuals with normal knees and with early and severe knee OA, providing data that have not been previously reported. The 5-year follow-up period also allowed us to determine the relationship between sHA and JSN, although no significant correlation with osteophyte development was found. This suggests that sHA concentration is a useful screening tool for detecting patients at risk of progressive JSN, even in individuals with normal or early OA of the knee.

The sHA concentration is reported to be a specific biomarker for OA of other major joints, besides the knee, and the lumbar spine [13], but sHA concentration increases with age because of an impaired ability to metabolize HA in the elderly [27]. HA is actively secreted into joint fluid by the surrounding synovial lining cells [28]. When intra-articular fluid pressure is increased, HA is also distributed to the liver (90 %), kidney (9 %), and spleen (1 %) via the lymphatic and capillary systems [27]. The increase in sHA concentration with age may even be a consequence of progressive age-related hepatic and renal impairment [29]. sHA concentration is reported to be influenced by other factors, including renal failure [30], liver failure [31], rheumatoid arthritis [32], malignancy [33], and physical activity [34]. We were able to adjust for these potentially confounding factors, allowing us to more accurately determine the relationship between sHA concentration and knee OA.

Changes in the femoro-tibial JSW are recommended as the primary assessment of joint damage in knee OA [35]. The results of studies that have sought to quantify the annual progression of JSN are affected by the population studied. Mazzuca et al. reported that the mean annual JSN ranged from 0.06 to 0.6 mm per year [36], and Pavelka et al. reported that JSN progressed by 0.4 mm over 2 years in patients diagnosed with knee OA [19]. The annual increase in the incidence of osteophyte formation or JSN in middle-aged subjects with radiographically identified KL grades 0 or 1 OA of the knee is reported to be 4 % [37]. Our present findings, based on a relatively unselected cohort, are in broad agreement with these studies.

We used the KOACAD system to measure JSW. The use of this validated technique permitted an objective analysis of an important outcome measure and removed the possibility of bias. JSW is correlated with cartilage volume in the medial femoro-tibial compartments [38], and progression of radiographic JSN appears to be predictive of cartilage loss as assessed with MRI [39]. Nevertheless, JSN should be evaluated very carefully on radiographs because it is affected by meniscal malposition and degeneration as well as cartilage defects [40]. Distinguishing between cartilage defects with meniscal tears and dislocation in those with painful early OA is important [41]. Six of our 450 participants (1.3 %) underwent total knee arthroplasty during the study period, broadly comparable with the findings of a cohort study conducted by Muraki et al., in which 30 people (1.0 %) in a population of 2,992 underwent knee arthroplasty during a 3.3-year surveillance period [42].

The present study has several limitations. First, we did not evaluate the development or progression of OA in other joints besides the knee, and the presence of generalized OA is reported to be associated with the progression of knee OA [43]. Furthermore, patients who have generalized OA at multiple sites, including the hands [44] and hip [11], also have higher sHA concentrations. Nonetheless, OA of the knee appears to have the greatest influence on sHA concentration. Inoue et al. found that the correlation between sHA concentration and OA was greatest in those with knee OA (*r* = 0.395), compared with OA of the hip (*r* = 0.108), or wrist and hand (*r* = 0.262) [12]. Although it would have been better to account for generalized OA in the sHA concentration, we could not examine radiographs of all the joints in each patient for this study. Second, the follow-up rate at the 5-year endpoint was only moderate because of a loss of participants. This presents a potential risk of selection bias. The sample number of the cohort was determined by the voluntary participation of community residents because this checkup was performed as part of an annual general health checkup; we could not control the follow-up rate. A large sample number is important for accurate statistics, and a high follow-up rate is also important for maintaining the characteristics of the subjects at baseline. We believe that the effect on the results of the patients dropping out was minimal because the characteristics of the followed subjects and those who dropped out were very similar. Third, there was a statistical limitation. The relationship between sHA and the progression of OA was assessed using two tests, linear regression analysis and logistic regression analysis. The concentration of sHA was a continuous parameter, which made it difficult to evaluate the outcomes using logistic regression analysis. Although it would have been better to use cutoff values or 10-unit shifts for this analysis, we did not want to statistically overestimate. Further, we could not use such methods because we found no past literature describing the creation of categories or use of such sHA scales that were based purely on evidence. Still, we found a significant correlation between sHA and progression of OA using logistic regression analysis, and the confidence interval was very tight. Although this was a limitation, the aims of the present study were to determine the relationship between sHA concentration and longitudinal changes in radiographic OA and the utility of using sHA to predict JSN, which were appropriately tested using linear regression analysis. Fourth, it was difficult to analyze the cutoff value for the development of OA with this study design. The cutoff values were 35.1 ng/ml for the development of OA, and 59.1 ng/ml for progression of OA. To better determine the cutoff value for the development of OA, subjects from a wider range of ages would be needed. Here, the patients were limited to middle-aged women and included many young patients at very low risk of developing OA. Furthermore, we discussed above why sHA concentration may vary with the severity of OA. The presence of OA is a risk factor for the acceleration of progressive OA. We believe that this point is one reason why the predictive value of sHA for progression was higher than that for OA development. Fifth, we were only able to measure sHA concentration, even though other biomarkers have been reported to be useful [45, 46]. Ishijima et al. reported that the serum concentrations of cartilage type II collagen cleaved by collagenase and cartilage type II procollagen carboxy propeptide, and the urinary concentration of cartilage type II collagen C-telopeptide, are elevated in patients with knee pain, in addition to the sHA concentration [14]. Although sHA is reported to have better predictive value for knee OA [7], the etiology of disease development and progression may vary between individuals and populations. The relationships between potential biomarkers and whether they complement each other warrant further study [47]. Sixth, the best method for measuring JSW using radiography remains controversial, and radiography-based values are typically less than those measured using MRI. In the present study, to achieve a more objective evaluation, each parameter was measured using the KOACAD system. Although the range of standard deviations was slightly wider than ideal, these results were similar to those measured by Oka et al., who reported mean JSWs of 3.22 ± 0.96 mm in male subjects and 2.65 ± 0.95 in female subjects based on 5,950 knee radiographs [48].

Despite these limitations, the results of this study based on a general population clearly show that sHA concentration is strongly associated with the severity of knee OA, progression of the disease, and the extent of knee symptoms. This longitudinal follow-up study elucidated the relationship between sHA concentration and radiographic changes in the knee over 5 years. sHA appears to be a valuable biomarker for knee OA and has utility as part of routine clinical evaluation to allow for early detection and intervention to prevent the progression of knee OA.

## Conclusions

The concentration of sHA was positively correlated with the severity of radiographic knee OA. Furthermore, over 5 years, higher sHA concentrations were positively correlated with the progression of KL grade in those with knee OA and of JSN in those both with and without knee OA. The cutoff values of sHA concentration predictive of the development and progression of OA were estimated. These results suggest that elevated sHA concentration can be used as a predictor of knee OA progression.

## Abbreviations

AUC: area under the curve
BMI: body mass index
HA: hyaluronic acid
HA: serum hyaluronic acid
JPEG: Joint Photographic Experts Group
JSN: joint space narrowing
JSW: joint space width
KL grade: Kellgren–Lawrence grade
KOACAD: knee osteoarthritis computer-aided diagnosis
MRI: magnetic resonance imaging
OA: osteoarthritis
ROC: receiver operating characteristic
